# Feasibility of stereotactic optical navigation for needle positioning in percutaneous nephrolithotomy

**DOI:** 10.1007/s00345-024-04870-0

**Published:** 2024-03-20

**Authors:** I. M. Spenkelink, X. Zhu, J. J. Fütterer, J. F. Langenhuijsen

**Affiliations:** 1 Department of Medical Imaging, Radboud University Medical Center, P.O. Box 9101, 6500 HB Nijmegen, The Netherlands; 2 Department of Urology, Radboud University Medical Center, Nijmegen, The Netherlands

**Keywords:** Percutaneous nephrolithotomy, Navigated needle positioning, CBCT imaging, Stereotactic navigation, Optical navigation

## Abstract

**Background:**

This study assessed the feasibility of acquiring single-attempt access to the pelvicalyceal system during percutaneous nephrolithotomy (PCNL) using stereotactic optical navigation combined with cone-beam CT (CBCT) imaging.

**Methods:**

Patients with a PCNL indication were prospectively included in this IRB approved study. After sterile preparation, fiducial markers were attached to patients’ skin. An initial intraprocedural CBCT scan was acquired, on which the urologist planned the needle trajectory using the navigation software. After verifying that no critical structures were crossed, the needle guide was aligned with the plan. A needle was manually inserted through the needle guide to the indicated depth and a second CBCT scan was performed for needle position confirmation. Both, scanning and needle insertion, were performed under apnea. The study evaluated technical success, accuracy, procedure time, complication rate, and radiation dose.

**Results:**

Between June 2022 and April 2023, seven patients were included. In all patients, the navigation system allowed safe puncture. However, the technical success rate was only 29%. In 42% of the cases, pelvicalyceal access was achieved by a small manual adjustment. In the remaining 29%, the needle was retracted and positioned per clinical standard. The average deviation between the needle and target was 5.9 ± 2.3 mm. The average total procedure time was 211 ± 44 min. The average radiation exposure was 6.4 mSv, with CBCT scanning contributing to 82% of this exposure.

**Conclusions:**

The optical navigation system facilitated safe needle insertion but did not consistently ensure accurate one-attempt needle positioning for PCNL. Real-time visualization and trajectory correction may improve the technical success rate.

## Introduction

The incidence and prevalence of kidney stones have been increasing over the last decades [1, 2]. According to EAU guidelines, percutaneous nephrolithotomy (PCNL) is the first-line treatment for large (≥ 20 mm) or complex renal stones [3]. An essential aspect of this treatment is acquiring access to the pelvicalyceal system, which is also the most challenging step [4–7]. This step is usually guided by ultrasonography, fluoroscopy, or a combination of both. Translating two-dimensional (2D) images to the three-dimensional (3D) anatomy of the pelvicalyceal system can be challenging, which could lead to repeated kidney punctures [8, 9]. Repeated puncturing is correlated with a 2.6-fold increase in odds of bleedings [10]. To address this issue, multiple technologies were developed to aid urologists in calyceal punctures, ranging from 3D models and holograms to intraprocedural 3D imaging and instrument guiding systems [11–13].

Optical navigation systems can assist in optimizing needle path planning based on 3D imaging and help specialists in precise guiding by means of a needle guide. Furthermore, using intraoperative cone-beam CT (CBCT) imaging in the hybrid operating room (OR), the procedure is planned without altering the position of the patient. This could improve needle positioning accuracy in the PCNL procedure. We hypothesized that single-attempt access through a calyx of choice, could improve the stone-free rate, which could justify potentially higher radiation doses in specific patients. Therefore, this study aimed to assess the feasibility of acquiring single-attempt access to the renal pelvicalyceal system using a stereotactic navigation system combined with CBCT imaging.

## Materials and methods

### Study design

This prospective feasibility study aimed to include ten adult patients with an indication for PCNL. The study was approved by the local medical ethical review board. All patients provided written informed consent. Exclusion criteria were: (1) an active urinary tract infection at the time of PCNL, (2) anatomical abnormalities that prevent safe PCNL access or surgery in prone position, (3) an absolute indication for continuation of anticoagulant medication, (4) presence of (potentially) malignant kidney tumors (5) pregnancy, and (6) age below 18 years.

### Stereotactic optical navigation system

In this study, the CAS-One IR system (CAScination AG, Bern, Switzerland) was used for stereotactic optical navigation. This system utilizes markers to track both the patient and the needle aiming device, enabling real-time patient-to-image registration. The system was originally developed for CT-guided ablations, where it showed high accuracy in probe positioning [14], but is also compatible with CBCT imaging [15].

### Training stereotactic navigation

Before the start of patient inclusion, a clinical application specialist of the company of the stereotactic optical navigation device trained the urologist. Multiple needle path planning and insertions were performed on a phantom (Quant, CAScination AG, Bern, Switzerland). Furthermore, a clinical application specialist was available during the first two patient cases.

### Navigated PCNL procedure

**Fig. 1:**
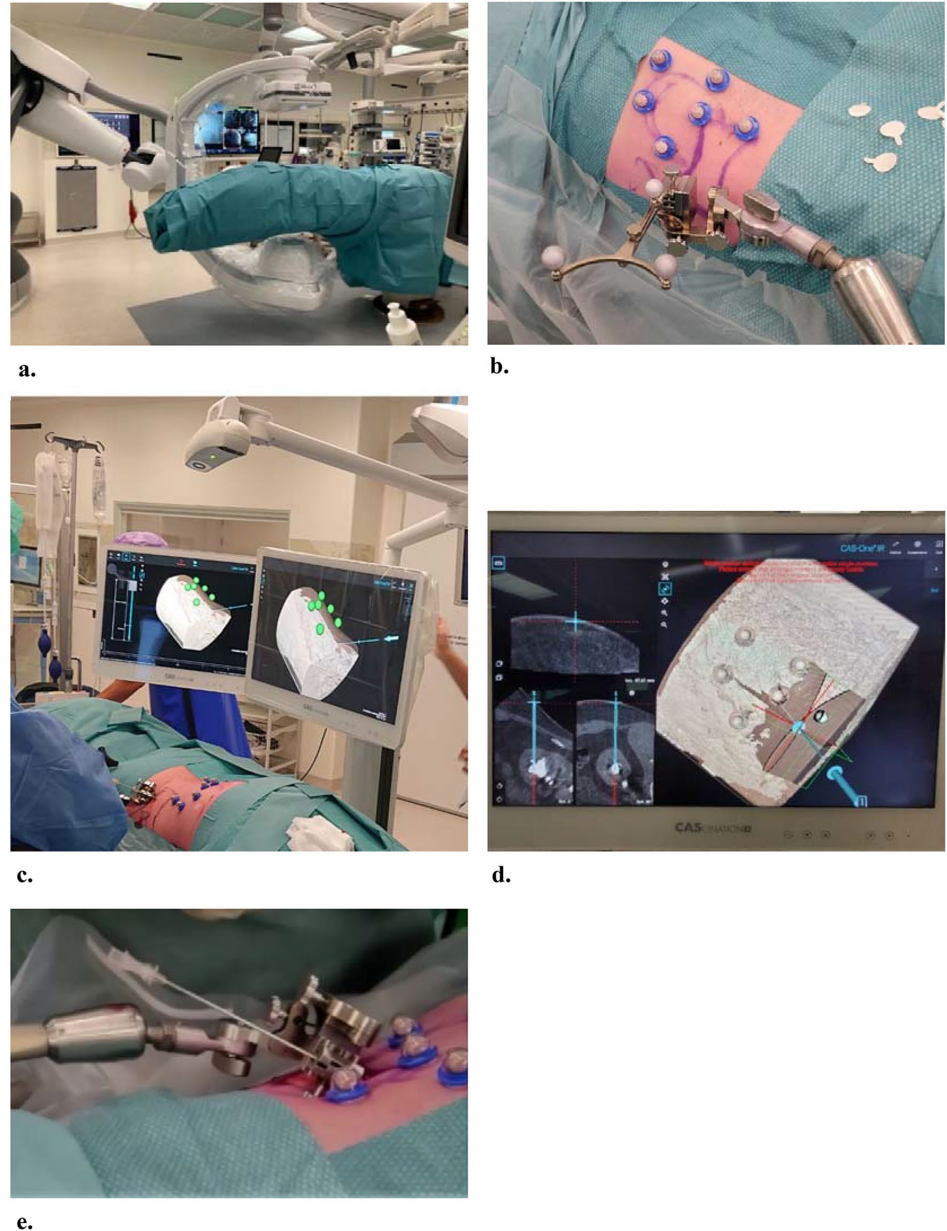
Stereotactic optical navigation for needle guidance in the percutaneous nephrolithotomy procedure. **a** Overview of the operating room with the patient prepared for cone-beam CT imaging. **b** Photograph of the fiducial markers on the skin, positioned around the expected needle entry point, and on the aiming device, which is positioned in the needle guide. These markers are used for image-to-patient registration. **c** Overview of the set-up for optical navigation with the CAS-One IR system. The camera detects the markers on the skin and correlates this with the cone-beam CT scan. The markers are shown as green spheres in the scan. **d** Using the navigation software, the needle tract is planned by selecting an entry point on skin level and a target point in the selected calyx. **e** After aligning the aiming device with the planned trajectory, the needle is inserted manually through the needle guide

All procedures were performed in a hybrid OR by a urologist experienced in PCNL, while the patient was under general anesthesia. After positioning of a ureteral catheter, patients were positioned in prone position on a radiolucent table. Following skin disinfection, they were fully draped in a circumferential manner (Fig. 1a). Unlike the standard PCNL procedure, no dilation of the renal collecting system was performed. Six fiducial marker spheres were attached to the skin of the patient around the intended puncture position (Fig. 1b). Ten minutes after intravenous injection of 70 mL contrast agent (Iomeron 300, Bracco Imaging Deutschland GmbH, Konstanz, Germany), a CBCT acquisition (ARTIS pheno, Siemens Healthineers, Forchheim, Germany) was performed to plan the needle trajectory. The trajectory was planned using CAS-One IR software by selecting the target and entry point (Fig. 1c, d). The resulting trajectory was assessed, and if required adjusted, to ensure that no risk structures were crossed. After confirming the trajectory, the aiming device was positioned accordingly, and an 18G needle (Cook Incorporated, IN, USA) was inserted manually (Fig. 1e). A second, control CBCT scan was performed to validate the needle location. Both CBCT scans and needle positioning were performed in expiratory apnea. If the intended calyx was punctured, the procedure was continued by dilating the tract as per clinical standard. If there was no access to the pelvicalyceal system, but the needle was close to a calyx, the needle was manipulated manually. If this was not possible, the needle was removed, and the procedure was performed as per clinical standard (i.e., using a combination of ultrasound and fluoroscopy in our institute). Six weeks after the procedure, patients were seen in the outpatient clinic to assess stone-free results and registering post-operative complications according to the Clavien–Dindo classification.

### Procedure adjustments

Based on evaluation of the first patient, we focused on improving the accuracy of needle positioning by minimizing kidney motion. This involved addressing two aspects: reducing motion between two instances of apnea and minimizing motion during the puncturing process. After onset of apnea, we waited 10 s after plateauing of the end-tidal carbon dioxide waveform to eliminate any motion due to passive expiration. We also attempted to refine our technique for puncturing the renal capsule using the pinch method and a 16 Gauge needle.

### Data collection

Demographic parameters were collected, including age, sex, and body-mass-index (BMI). Stone load, location, size, and STONE scores were obtained based on a pre-operative CT-scan. The following data regarding the PCNL procedure were collected: technical success, procedure time, perioperative complications, radiation exposure, and existence of residual stone fragments. Technical success was defined as gaining access to the intended calyx. The total procedure time was further divided into: patient preparation time, surgical preparation time, planning time, and needle positioning time. Furthermore, technical data to assess the accuracy were obtained from CAS-One IR’s log file. Accuracy of the needle positioning was assessed in terms of Euclidean, lateral and depth distance between the needle tip and the planned target, and angular deviation between the needle and the planned path.

### Statistical analysis

Descriptive statistics were calculated to present patient characteristics and outcome data as mean ± standard deviation (SD). Statistical calculations were performed using the Statistical Package for the Social Sciences version 25.0 (SPSS, IBM, New York, USA).

## Results

Between June 2022 and April 2023, 7 out of 10 patients were included. Due to the low technical success rate, the study was ended prematurely. Table 1 shows the patient characteristics. No adverse events occurred during this study. The mean total procedure time was 211 ± 44 min. Patient preparation time, surgical preparation time, planning time, and positioning time were 17 ± 16 min, 58 ± 19 min, 25 ± 5 min, and 5 ± 5 min, respectively.

**Table 1:** Baseline patient and stone characteristics

Patient characteristics	*n* = 7
Male/female	5/2
Age (years), mean ± SD	53 ± 18
BMI (kg/m^2^), mean ± SD	26.5 ± 3.6
Kidney left/right, *n* (%)	4 (57)/3 (43)
Main stone location, *n* (%)	
Upper calyx	2 (29)
Middle calyx	1 (14
Lower calyx	2 (29)
Pelvis	2 (29)
Non-staghorn	5 (71)
Staghorn	2 (29)
Stone size (mm), mean ± SD	29 ± 12
STONE score risk group, *n* (%)	
Low	7 (100)
Moderate	0 (0)
High	0 (0)
ASA physical status, *n* (%)	
1	1 (14)
2	5 (71)
3	1 (14)

*SD* Standard deviation, *BMI* body-mass-index, *ASA* American Society of Anaesthesiology

Needles were positioned over a mean needle tract length of 10.7 ± 2.4 cm. The distances between the needle tip and the planned target were 5.9 ± 2.3 mm, 5.3 ± 2.3 mm, and 1.8 ± 2.1 mm in Euclidian, lateral and depth directions, respectively. The angular deviation between the needle and the planned trajectory was 2.5 ± 2.3°.

Adjusting our apnea technique reduced motion between two apnea periods as could be seen on the overlay of the pre- and post-insertion CBCT scans. However, our attempts to improve the piercing of the renal capsule had limited impact on the motion of the kidney. In some patients punctured with the 18G needle, a slight bend in the needle was noticed on the CBCT scan. This could have occurred when the needle passes from tissue with a low stiffness to a higher stiffness, for example at the renal capsule.

The total average patient radiation dose was 6.4 mSv. The 3D CBCT scan contributed to 5.1 mSv (82%), while fluoroscopy accounted for 1.0 mSv (18%) of radiation exposure. At the end of the procedure, a stone-free status was expected in 71% of the patients. At 6 weeks after the procedure, the overall stone-free rate was 43%. The results are summarized in Table 2.

**Table 2:** Percutaneous nephrolithotomy procedure parameters

Technical success, *n* (%)	2 (29)
Procedure time (min), mean ± SD	
Anesthesia preparation	17 ± 16
Surgical preparation	58 ± 19
Planning	25 ± 5
Needle positioning	5 ± 5
Total procedure	211 ± 44
Needle tract length (mm), mean ± SD	107 ± 24
Accuracy of needle positioning	
Euclidean (mm), mean ± SD	5.9 ± 2.3
Lateral (mm), mean ± SD	5.3 ± 2.2
Depth (mm), mean ± SD	1.8 ± 2.0
Angular (degree), mean ± SD	2.5 ± 2.3
Radiation dose (mSv), mean ± SD	6.4 ± 2.9
Stone-free rate, *n* (%)	3 (43)
Surgical complications, *n* (%)	0 (0)
Postoperative hospital time (d), mean ± SD	3 ± 2
Pre-operative hematocrit	0.42 ± 0.07
Post-operative hematocrit	0.39 ± 0.08

*SD* Standard deviation

## Discussion

This study showed that the stereotactic optical navigation system could be used to plan the needle path and safely insert the needle. However, the deviation between the needle and the target of 5.9 mm was not sufficiently accurate for the PCNL procedure. The low technical success of 29%, together with prolonged procedure times of 211 min and a patient radiation dose of 6.4 mSv resulted in the decision to prematurely end the study, even though no adverse events occurred.

The low technical success rate may be attributed to the lack of real-time feedback on needle and target positions. The retroperitoneal position and the high stiffness of its capsule allow easy movement of the kidney during needle manipulation [16], which can go unnoticed without real-time imaging. Conventional techniques like fluoroscopy and ultrasound provide this real-time feedback, enabling immediate needle position adjustments in case of respiratory movement and tissue deformation. Incorporating hydronephrosis possibly could have enhanced the technical success rate. However, only in complex anatomical cases, the additional procedure time and radiation dose might be justified. In such scenarios, a high intrinsic accuracy of the system is crucial for the success of the procedure.

The surgical preparation time accounted for 27% of the total procedure time, primarily due to the elongation of lines necessary for anesthesia and patient monitoring and preparing the patient and the c-arm for acquiring CBCT scans. Although achieving proficiency through a learning curve could potentially shorten the duration, this is always an extra step when using the CAS-One IR system. Notably, our study did not observe any reduction the surgical preparation time. In addition, contrast agent administration was required to visualize the pelvicalyceal system on CBCT scans, which can affect renal function [17]. Therefore, the navigated needle positioning had drawbacks for the study patients, while no significant benefits were evident in this study.

While this is the first study to assess the feasibility of using the CAS-One IR system for navigated needle positioning in the PCNL procedure, others have used the CAS-One IR system for needle positioning in liver tumors. These studies have reported mean Euclidean, lateral, depth, and angular errors ranging from 3.7 to 5.8 mm, 2.2 to 4.0 mm, 1.3 to 3.4 mm, and 1.8 to 2.7°, respectively [14, 18–20]. These findings are comparable to our results. Only our lateral deviation is higher than previously reported deviations, which could be explained by our long needle tract lengths of 107 mm.

Various studies have explored different needle guidance methods for PCNL procedures. Jiao and Ritter also used c-arm CBCT imaging, however, without additional navigation techniques [21, 22]. Using the c-arm laser, they achieved successful needle placement on the first attempt in 66.6 and 80% of cases, respectively. They also concluded that kidney motion negatively affected the accuracy of needle positioning. Another method, iPad-assisted needle puncture, relied on pre-operative CT markers to align 3D anatomy with the patient intraoperatively via an iPad [23]. This study reported a single puncture technical success of 64%. However, in addition to be impaired by kidney motion, this technique also lacked intra-operative visualization of the surrounding organs to ensure a safe needle track. Electromagnetic (EM) tracking alone also lacks visualization of surrounding organs. However, studies combining EM guidance with ultrasound reported technical success rates of 100%, which could be the result of a real-time visualization of the needle in the ultrasound transducer plane and an estimation of the needle position out-of-plane [11, 12]. Using this technique, one EM probe is positioned in the targeted calyx and the other one is in the needle tip. A possible challenge in obese patients is the signal intensity dependence on the distance between probes.

In addition, robots were evaluated for aiding needle positioning in PCNL. The percutaneous access to the kidney with the remote center of motion device (PAKY–RCM) is a table mounted system that uses fluoroscopy imaging for path planning and monitoring of needle progression. On average, 2.2 attempts were needed, achieving access in 87% of patients [24]. The Automated Needle Targeting with X-ray (ANT-X) robot, a patient mounted system, also uses fluoroscopy for needle guidance. Furthermore, it uses artificial intelligence to automate the needle puncture trajectory based on the fluoroscopy images. It achieved access in all patients, with a single-puncture success rate of 50%, and a 100% success rate in an average of 1.8 needle punctures [25]. Although not yet clinically deployable, these techniques show promise in assisting clinicians with needle positioning in PCNL.

Limitations of this study include the small number of included patients, which might restrict the ability to undergo a complete learning curve. Due to high demand of the use of the hybrid OR we had limited access to this facility. Furthermore, despite efforts to optimize the protocol, including needle thickness adjustments, direct renal capsule puncture, and ensuring no needle tension during skin penetration, these measures did not improve accuracy or enable direct access attainment. A limitation of using the stereotactic navigation system was the requirement for two 3D scans for path planning and needle position confirmation, resulting in a high patient radiation dose. In addition, real-time visualization was not performed and feedback on the needle position was only obtained through the second 3D CBCT scan.

To help clinicians in selecting the optimal approach, studies that compare existing techniques that aid needle puncture in PCNL procedures are essential, evaluating accuracy, procedural time, radiation exposure, and patient outcomes. Furthermore, research on new systems should focus on techniques that provide real-time feedback. A system that accounts for (respiratory) motion and tissue deformation, while visualizing neighboring organs and ensuring a zero or low radiation dose would be optimal. However, costs and applicability of such a system also must be taken into account.

## Conclusion

While safe needle insertion with the stereotactic optical navigation system was feasible, the system could not ensure accurate needle positioning inside the pelvicalyceal system in the PCNL procedure within a single attempt, suggesting the device’s suboptimal suitability for this procedure. The absence of real-time visualization during needle insertion, which hindered adaptive corrections for deviations in needle direction or target movement, likely contributed to the low technical success rate observed in this study.

## Data Availability

Data and materials are not publicly available, but are available by the corresponding author upon reasonable request.
